# A Micro-Nodal Tungsten-Rhenium Thin-Film Thermocouple Based on Electrohydrodynamic Printing

**DOI:** 10.3390/ma18051031

**Published:** 2025-02-26

**Authors:** Shuntao Hu, Jun Chen, Shigui Gong, Ying Li, Shilong Liu, Jihao Li, Shuaida Wang, Zhenyin Hai, Zhichun Liu, Junyang Li

**Affiliations:** 1 School of Electronic Engineering, Ocean University of China, Qingdao 266100, China; hushuntao@stu.ouc.edu.cn (S.H.); chenjun7942@stu.ouc.edu.cn (J.C.); lsl3288@stu.ouc.edu.cn (S.L.); lijihao@stu.ouc.edu.cn (J.L.); wangshuaida@stu.ouc.edu.cn (S.W.); 2 School of Opto-Electronic and Communication Engineering, Xiamen University of Technology, Xiamen 361005, China; 2322101004@stu.xmut.edu.cn; 3 School of Mechanical and Electrical Engineering, Shenzhen Polytechnic University, Shenzhen 518055, China; 4 School of Aerospace Engineering, Xiamen University, Xiamen 361005, China

**Keywords:** electrohydrodynamic printing, thin-film thermocouples, response time, measurement accuracy

## Abstract

High-temperature thin-film thermocouples (TFTCs) have gained significant attention in the aerospace and energy industries due to their compact size and millisecond response time. Although previous studies have reduced the size of TFTCs to the millimeter scale, the heat flow field has continued to limit temperature measurement accuracy. To address this issue, this study used an electrohydrodynamic printing process to fabricate tungsten-rhenium TFTCs with a thickness at the micrometer scale. In the static test, the tungsten-rhenium TFTCs showed good performance with a measurement accuracy better than 1.2%, repeatability better than 0.99%, and a drift rate of 0.72%/h. In dynamic tests, the response time was 1.2 ms. Additionally, during flame gun heating tests, the response time and temperature measurement accuracy exceeded those of the standard thermocouple.

## 1. Introduction

The miniaturization of high-temperature sensors is of great significance in the aerospace, iron and steel metallurgy, and energy industries [1,2,3], as it not only reduces the weight of the equipment and saves space but also significantly improves the measurement accuracy and response time, especially in complex environments, such as high temperatures, high pressures, or strong vibration conditions, that can show higher adaptability [4]. For example, miniaturized temperature sensors are capable of in situ measurement of critical engine components (e.g., turbine, combustion chamber) to achieve real-time and accurate temperature monitoring, which supports the safe operation and efficient performance of the aircraft [5]. However, previous methods of high-temperature measurement, such as fiber optic thermometer [6], infrared thermometer [7], and thermistor [8], fail to simultaneously meet the demand for small-volume, real-time, and fast high-temperature measurement. Therefore, more and more researchers have recognized the need for sensor miniaturization.

In order to meet the demand for miniaturized temperature sensors with fast response, thin-film thermocouples have been widely studied because of their small size, high accuracy, and fast response [9]. Currently, the main methods for manufacturing TFTCs are screen printing, magnetron sputtering, and other methods. Screen printing is a process that utilizes a screen as a template and prints ink or paste pressed through the mesh onto the substrate surface by means of a squeegee [10]. It is an important thick-film manufacturing technology widely used in electronics, printing, and manufacturing. Screen printing has the advantages of a simple process and lower cost, but it has limited manufacturing resolution and poor film thickness uniformity [11,12]. A tungsten-rhenium thin-film thermocouple prepared on an alumina substrate using screen printing achieved an overall thickness of 1 mm with a response time of 1.74 s by Su et al. [13]. Fan et al. prepared thermocouple probes with a maximum temperature limit of 1200 °C using screen printing [14]. Magnetron sputtering is a highly efficient and controllable thin-film deposition technique suitable for a broad range of materials, particularly excelling in applications that require high-quality films and low-temperature deposition. It has widespread applications in semiconductor, optics, and materials science [15]. Magnetron sputtering is characterized by good film adhesion and excellent uniformity, but has the disadvantages of high equipment cost, slow processing, time consuming, and limited rapid high-volume preparation. Sensor failure due to oxidation-induced horizontal diffusion at 620 °C in tungsten-rhenium TFTCs fabricated by magnetron sputtering was reported by Ruan et al. [16]. Liu et al. fabricated ITO/Pt, In_2_O_3_/Pt, and ITO/In_2_O_3_ thermocouples via magnetron sputtering and investigated their performance differences at various annealing temperatures [17]. In contrast the electrohydrodynamic atomized printing process provides an efficient, flexible, and sustainable technological path for the preparation of thin-film thermocouples with its high precision, low cost, and wide applicability [18,19,20]. Electrohydrodynamic atomized printing is an advanced manufacturing technology that uses electric field force to drive the atomization of a liquid sprayed at a nozzle onto a substrate to form a pattern. The principle behind this process involves applying a high voltage between the nozzle and the receiving surface, causing the liquid to be stretched and ruptured by the electric field, which results in the formation of tiny droplets or atomized particles. These particles are then sprayed onto the target surface and are commonly used in applications, such as coatings, inkjet printing, and drug delivery [21,22,23]. The strength of the electric field and the properties of the liquid together determine the size, distribution, and direction of the atomized particles, making this technology particularly suited for precise control of droplet size and morphology. This phenomenon enables the precise ejection of nanometer-sized droplets, which results in the precise deposition of material on the substrate to form the desired film or microstructure [24,25].

In this work, we used an atomization process of electrohydrodynamic printing to process the TFTC on an alumina substrate of 100 μm thickness. Compared to other sensitive layer materials, tungsten and rhenium offer advantages, such as lower cost, high melting point, and excellent high-temperature stability, making them ideal choices as the sensitive layer material for the sensor. The effect of different sintering temperatures on the performance of tungsten-rhenium TFTCs was systematically investigated, and both dynamic and static tests were performed at the optimal sintering temperature. The tungsten-rhenium TCFC was then subjected to static tests in a high-temperature tube furnace, including fitting error, repeatability, and accuracy. Then, the dynamic test was carried out using a laser, and the response time of tungsten-rhenium TCFC reached 1.9 ms. Finally, the tungsten-rhenium TFTC was heated in air using a butane flame gun, and the results showed that its temperature measurement accuracy and response time were better than that of the commercial k-type thermocouple.

The structure of the article is organized as follows: Section 1: An introduction to the preparation process of thin-film sensors and an overview of the main contributions of this paper. Section 2: An introduction to the structure and fabrication method of the sensor. Section 3: Analysis and testing of the sensors. Section 4: Conclusions.

## 2. Materials and Methods

### 2.1. Materials

The tungsten paste and diluent used to prepare the tungsten-rhenium TFTC in this experiment were purchased from Shenzhen Saiya Electronic Paste Science and Technology Co., Ltd. (Shenzhen, China). Rhenium powder with a particle size of 10 μm was purchased from Shanghai Ruidong Chemical Group Co. (Shanghai, China). The Al_2_O_3_ substrate and Al_2_O_3_ discs were obtained from Dongfu Electronic Shopping Center (Dongguan, China). Tungsten wire and tungsten 74 wt%-rhenium 26 wt% wire, used for electrical signal transmission, were supplied by Qinghe Tengfeng Metal Materials Co. (Xingtai, China). The GSL-1750X high-temperature tube furnace was purchased from Hefei Kejing Material Technology Co. (Hefei, China). The B-type and K-type thermocouples used in the tests were obtained from Xinghua Shunsheng Electric Co. (Hefei, China). 

### 2.2. Electrohydrodynamic Printing System

**Figure 1:**
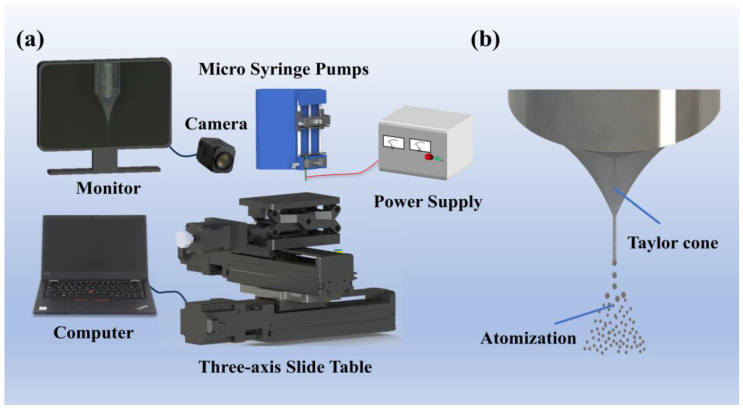
(**a**) Schematic diagram of a self-built electrohydrodynamic printing platform; (**b**) Schematic diagram of atomized jet.

**Figure 2:**
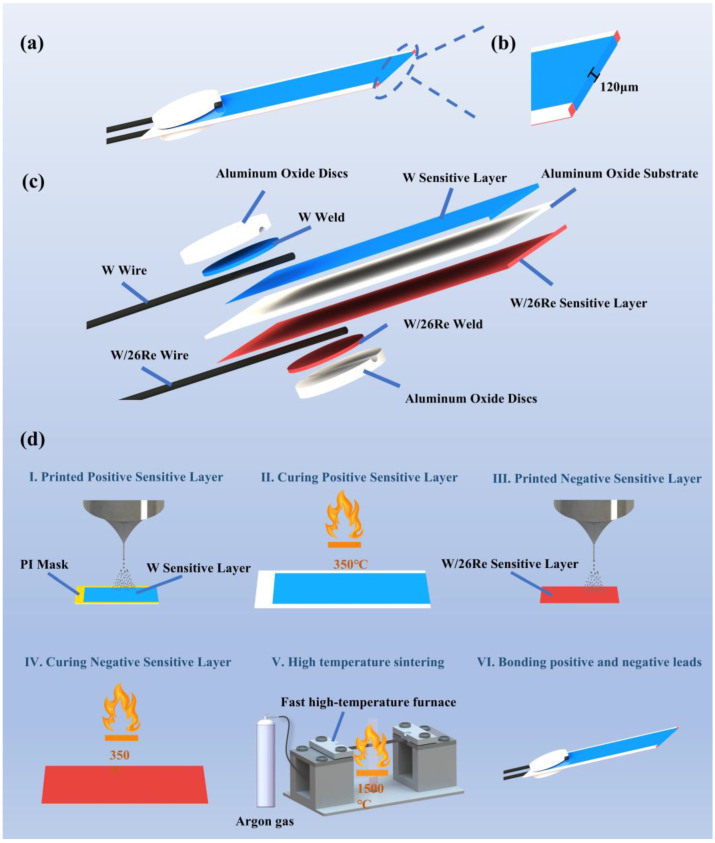
(**a**) Overall structure of the tungsten-rhenium TFTC; (**b**) Schematic diagram of the thermal node; (**c**) Explosion perspective of the tungsten-rhenium TFTC; (**d**) Manufacturing process of the tungsten-rhenium TFTC.

The schematic diagram of the self-constructed electrohydrodynamic printing system is shown in Figure 1a. It includes a programmable three-dimensional motion platform, a micro syringe pump, a metal needle with an inner diameter of 0.7 mm, a high-voltage power supply, a computer, and a micro-imaging acquisition module. The high resolution of the three-axis motion stage ensures precise movement of the substrate, with a resolution of 1.25 µm in the X- and Y-axes and 0.375 µm in the Z-axis. The metal needle is fixed to the syringe pump and connected to the high-voltage power supply. Syringe pumps are capable of delivering liquid at a minimum flow rate of 1.02 pL/min. As shown in Figure 2b, the combination of electric field forces, surface tension, viscous forces, and hydrostatic pressure creates a stable Taylor cone and a nanoscale ultrafine spray. By adjusting the camera angle, the conical jet at the needle tip is captured, and it is eventually deposited onto the substrate to form a continuous film [26].

### 2.3. Structure and Production

The overall structure of the tungsten-rhenium TCFC is shown schematically in Figure 2a, while the exploded view is presented in Figure 2c. The tungsten-rhenium TCFC consists of five main components: the alumina substrate, W and WRe26 films (positive and negative), W and WRe26 leads, W and WRe26 solder joints, and alumina discs. Positive and negative films are deposited on both sides of the alumina substrate, and the films are connected at the end face of the 100 µm thick alumina substrate to form a thermal node. The structure of the thermal node is shown in Figure 2b. Based on the Seeback effect, when the thermal nodes are at different temperatures, the free electrons in the metal conductor move due to the temperature gradient, generating a potential difference. This electric potential is proportional to the temperature difference, and by measuring the potential at both ends of the thermocouple, the temperature of the hot junction can be determined [27,28]. The alumina substrate has dimensions of 8 mm × 3 mm × 100 µm, the filament diameter of the W and WRe26 leads is 100 µm, and the diameter of the alumina discs is 2.5 mm.

The schematic diagram of the electrofluidic spray printing process for preparing the tungsten-rhenium TFTC is shown in Figure 2d. First, a mask was applied to one side of the alumina substrate using PI tape. Ultrasonic cleaning of alumina substrates with alcohol, acetone, and deionized water prior to printing. The positive paste consisted of tungsten paste and diluent in a 10:1 ratio, while the negative paste comprised tungsten paste, rhenium powder, and diluent in a 74:26:10 ratio.

Subsequently, the positive W film was printed using a micro syringe pump that supplied a constant flow rate of 6 µL/min. The height of the metal needle from the alumina substrate was set at 7–9 mm, the high-voltage power supply was set to 3.9–4.2 kV, and the movement rate of the three-dimensional motion platform was set to 3 mm/s. After the anodic W film was printed to a dense and uniform state using these parameters, it was placed on a digital display heating plate and cured at 350 °C for 20 min to remove organic components and prevent film rupture. Subsequently, negative WRe26 was printed on the opposite side of the alumina substrate using the same parameters and cured under the same conditions. At the end of the curing process, the TFTC was placed in an ultrafast high-temperature furnace for flash sintering. Flash sintering technology utilizes the Joule heat generated by the electric current flowing through the sample to directly heat the entire sample, applying all the heat converted from electrical energy to the sintering process, significantly reducing energy dissipation. Flash Joule heating offers several advantages, including short sintering times, fast densification, no need for sintering additives, and suppression of oxidation, phase changes, and grain growth during sintering [29]. The sintering parameters were set to increase the temperature to 1500 °C within 5 s, hold it for 180 s, and then reduce the temperature to room temperature within 30 s to complete the sintering. The entire sintering process was conducted under an argon atmosphere. After sintering, the thickness of the monolayer film was approximately 10 µm, and the overall thickness of the TCFC was around 120 µm.

## 3. Results and Discussion

### 3.1. Effect of Sintering Temperature on TFTC

**Figure 3:**
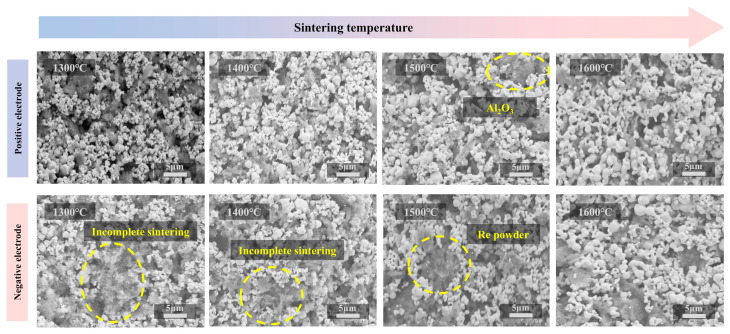
Characterization of surface morphology at different sintering temperatures.

To investigate the effect of ultrafast high-temperature furnace heat treatment on the thermoelectric properties of tungsten-rhenium TFTC, the effect of different sintering temperatures on tungsten-rhenium TFTCs was investigated. As shown in Figure 3, the microstructure of the positive and negative films of the tungsten-rhenium TFTC was observed by SEM at sintering temperatures ranging from 1300 °C to 1600 °C. The results revealed that, with increasing sintering temperature, the grain size of the films increased. However, the overall trend aligned with the inhibition of grain growth during flash sintering [29]. At sintering temperatures of 1300 °C and 1400 °C, incomplete sintering was observed in the tungsten-rhenium TFTC.

**Figure 4:**
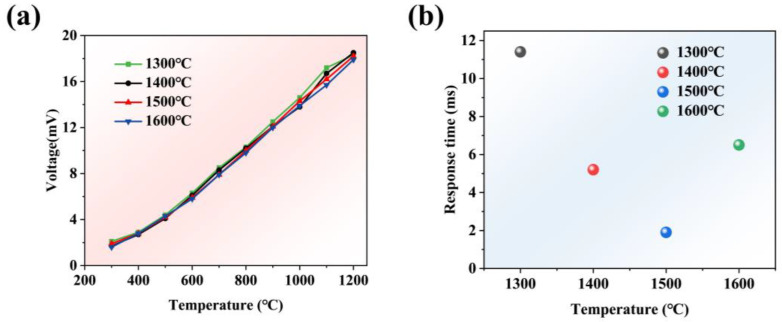
(**a**) Voltage curve at four different sintering temperatures. (**b**) Dynamic response results at four sintering temperatures.

Figure 4a shows the heating curve of the tungsten-rhenium TFTC sintered at temperatures of 1300 °C, 1400 °C, 1500 °C, and 1600 °C, with the temperature rising from 300 °C to 1200 °C. It can be observed that, at sintering temperatures of 1300 °C and 1400 °C, the curve fluctuates when the temperature exceeds 1000 °C, indicating incomplete sintering of the films, followed by sintering during the temperature increase. Figure 4b presents the dynamic performance results of the tungsten-rhenium TFTC at different sintering temperatures. The results show that the response times of the tungsten-rhenium TFTC at sintering temperatures of 1300 °C, 1400 °C, 1500 °C, and 1600 °C were 11.4 ms, 5.2 ms, 1.9 ms, and 6.5 ms, respectively, indicating that the tungsten-rhenium TFTC performs well at sintering temperatures above 1500 °C [13].

### 3.2. Sensor Performance Test

**Figure 5:**
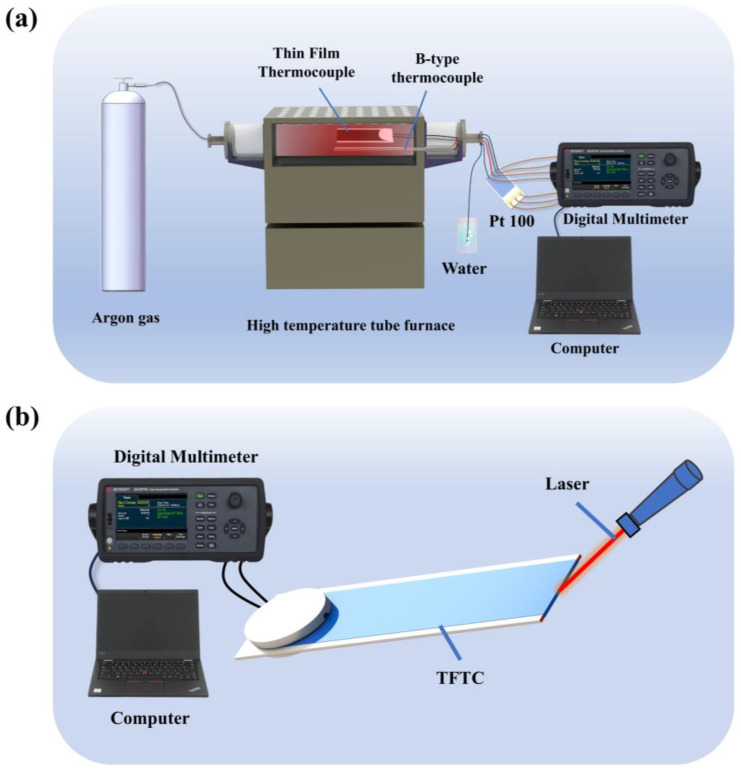
(**a**) Schematic diagram of the static test platform; (**b**) Schematic diagram of the dynamic test platform.

To conduct static testing of the tungsten-rhenium TFTC system, the static test platform shown in Figure 5a was utilized. The platform primarily consists of a high-temperature tube furnace, a digital multimeter, a Type B thermocouple, a three-wire platinum resistance temperature sensor, a computer, an argon gas cylinder, and a water cup. The hot end of the tungsten-rhenium TFTC and the Type B thermocouple being tested are placed in the constant-temperature zone of the high-temperature tube furnace, while the cold end is connected to the three-wire platinum resistance sensor. The three-wire platinum resistance sensor is connected to the digital multimeter via a copper wire, which also connects the cold end to the multimeter. Temperature data from both the cold end and the hot end are recorded and saved on the computer. Before the test, the valve of the argon gas cylinder is opened to fill the tube furnace with argon gas. A steady stream of bubbles appearing in the water cup confirms that the gas flow is uniform. The desired temperature is then set using the temperature control program of the tube furnace. Tungsten-rhenium metal oxidizes at high temperatures in air, which is why an inert gas atmosphere is maintained in the furnace to prevent sensor oxidation [30].

The dynamic testing setup for the tungsten-rhenium TFTC is shown in Figure 5b. The platform primarily consists of a digital multimeter, a computer, and a laser heat source. A laser generator is used to transiently heat the hot end of the tungsten-rhenium TFTC.

**Figure 6:**
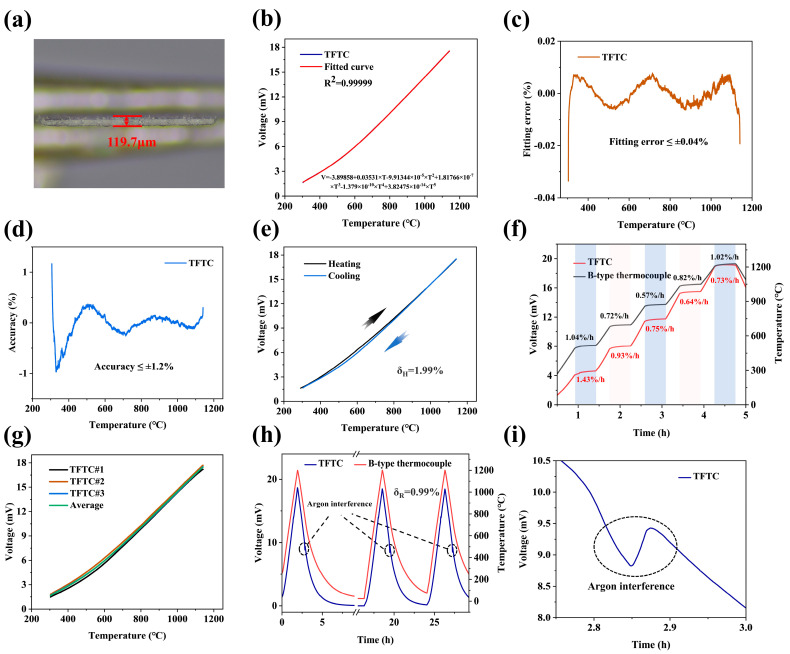
(**a**) Thermal nodes of the tungsten-rhenium TFTC under an optical microscope. (**b**) Voltage and fitting curves. (**c**) Fitting error of the measured values. (**d**) Accuracy error curve. (**e**) Single rise and fall temperature curve. (**f**) Five temperature step tests. (**g**) Consistency curves. (**h**) Three-cycle rise and fall temperature curve. (**i**) Voltage curve of tungsten-rhenium TFTC under argon interference.

Figure 6a shows the image of the TFTC thermal node under an optical microscope. To evaluate the potential performance of the sensors in real-world environments, static calibration tests were conducted over the temperature range of 300 °C to 1200 °C using the static test rig described earlier. The tungsten-rhenium TFTC was first heated from 300 °C to 1200 °C, and its voltage profile was recorded, as shown in Figure 6b. A fifth-degree polynomial was then fitted to the sensor data, achieving an effective model with an R^2^ value of 0.99999. The corresponding fitting error curve is presented in Figure 6c, showing a fitting error of less than 0.04%. Figure 6d illustrates the accuracy curve of the tungsten-rhenium TFTC over the temperature range of 300 °C to 1200 °C, with results indicating a temperature measurement accuracy better than ±1.2%. Figure 6e shows the single lift temperature profile of tungsten-rhenium TFTC, showing a hysteresis of less than 1.99%.

To verify the short-term accuracy of the tungsten-rhenium TFTC, it was further calibrated for five temperature segments, as shown in Figure 6f. The measured sensor sensitivity was 15.75 μV/°C, with a drift rate significantly better than that of Type B thermocouples in high-temperature ranges [31]. Consistency tests were also conducted on the same batch of sensors, and the results, as shown in Figure 6g, confirmed that the tungsten-rhenium TFTCs prepared using the electrofluidic imprinting method exhibit excellent consistency. Figure 6h presents three rounds of repeatability tests conducted on the same tungsten-rhenium TFTC. The results indicate that the sensor follows the same heating and cooling trends as the Type B thermocouple, with a repeatability error of less than 0.99% across the three rounds. During cooling to 750 °C in each round, the flow rate of argon in the tube furnace was increased. Since the argon temperature was significantly lower than that inside the tube furnace, this caused temporary interference in the sensor’s temperature measurements. Figure 6i shows a magnified view of the argon interference observed in Figure 6h. The thermoelectric potential of the tungsten-rhenium TFTC initially decreased due to the low-temperature argon but returned to normal after a short period. In contrast, no argon interference was detected by the Type B thermocouple.

**Figure 7:**
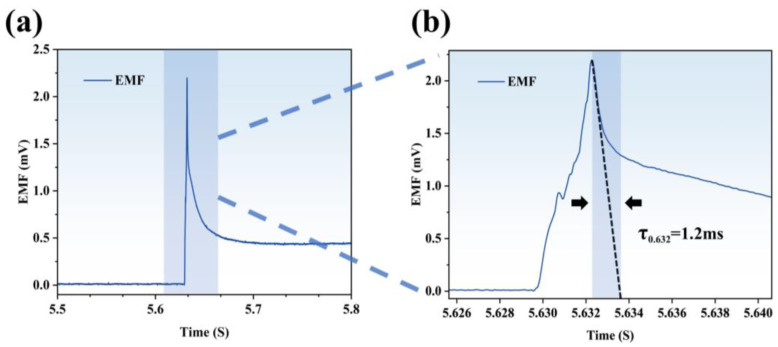
(**a**) Response time result of the tungsten-rhenium TFTC. (**b**) Specific response time results.

In dynamic testing, the laser generator parameters were set to a voltage of 200 V, a pulse width of 2.5 ms, and a serial frequency of 9 Hz. The test results are presented in Figure 7a, with Figure 7b showing a magnified view of the localized response. The result shows that the response time of tungsten-rhenium TFTC is 1.2 ms.

**Figure 8:**
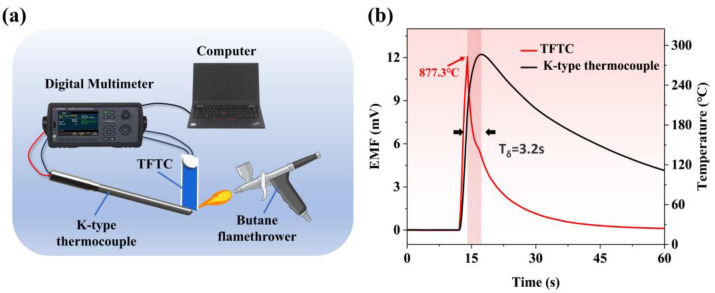
(**a**) Schematic diagram of dynamic butane flame test platform. (**b**) Real-time temperature response of TFTC and type k thermocouples under butane flame.

As shown in Figure 8a, a butane flame gun was used to heat the tungsten-rhenium TFTC and a K-type thermocouple for a short period [32]. Since the heat transfer effect is inversely proportional to the size of the thermocouple junction, a smaller thermocouple node results in a faster dynamic response [33]. The experimental results, presented in Figure 8b, demonstrated that the tungsten-rhenium TFTC achieved its maximum response 3.2 s faster than the K-type thermocouple. Furthermore, the tungsten-rhenium TFTC reached a maximum voltage of 12.05 mV, which corresponds to a temperature of 877.3 °C, as determined by the fitting formula. This result aligns with the short-term heating capability of the butane flame gun. In contrast, the K-type thermocouple detected a maximum temperature of only 286 °C under the same conditions. These findings confirm that the tungsten-rhenium TFTC exhibits superior dynamic response time and short-term temperature measurement accuracy compared to the K-type thermocouple.

## 4. Conclusions

In conclusion, we successfully developed a miniature tungsten-rhenium thin film thermocouple using electrohydrodynamic printing and fabricated thermal nodes on 100 μm thick alumina substrates through the atomization mode of electrohydrodynamic printing. The influence of various sintering temperatures on the performance of tungsten-rhenium TFTCs was systematically examined. The results indicate that these thermocouples exhibit excellent performance at sintering temperatures exceeding 1500 °C. In static tests, the TFTCs demonstrated an accuracy better than ±1.2%, repeatability better than 0.99%, and a drift rate of 0.73%/h at 1200 °C. In dynamic tests, the response time was 1.2 ms, and both temperature measurement accuracy and response time outperformed those of the K-type thermocouple. Additionally, during signal transmission from the thermocouple to the measuring device, electrical interference and noise may occur [34]. In the future, we will investigate the strength of the solder joints and the role of the protective layer in safeguarding the film to further enhance the electrical connection performance.

## Data Availability

The datasets presented in this article are not readily available because technical limitations. Requests to access the datasets should be directed to hst11104@163.com.

## Abbreviation

TFTC	Thin-Film Thermocouple
